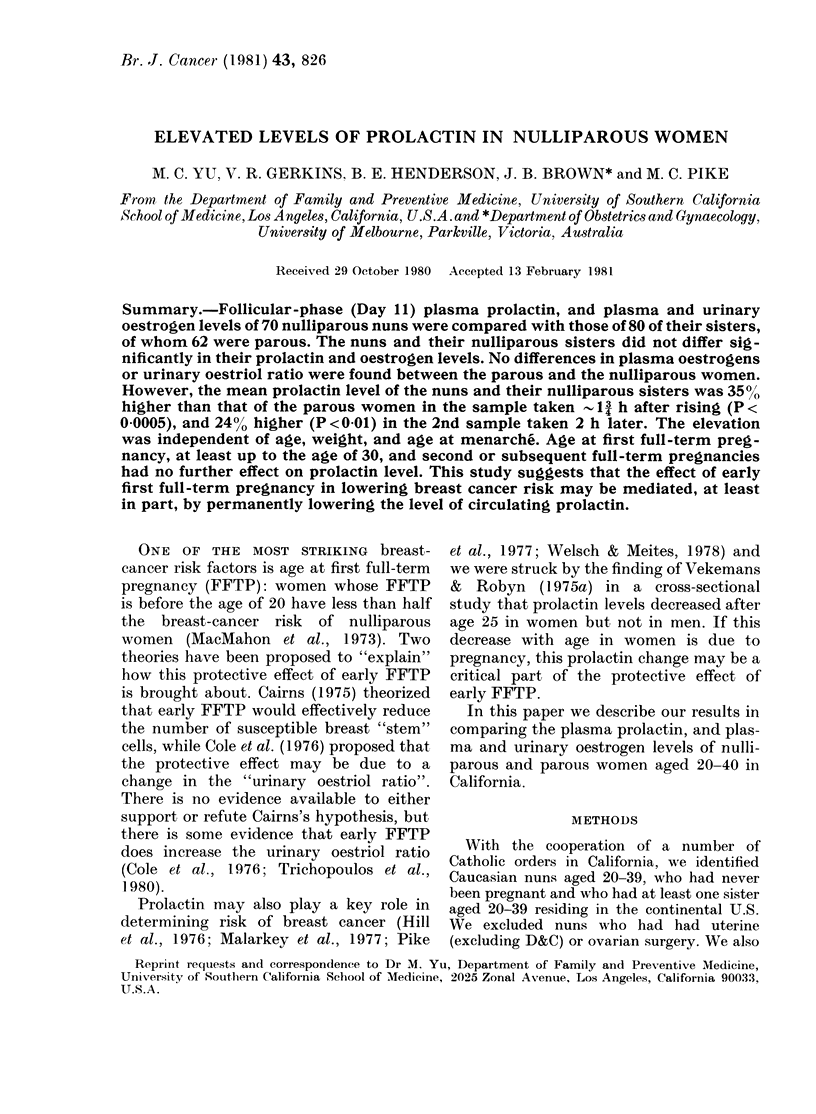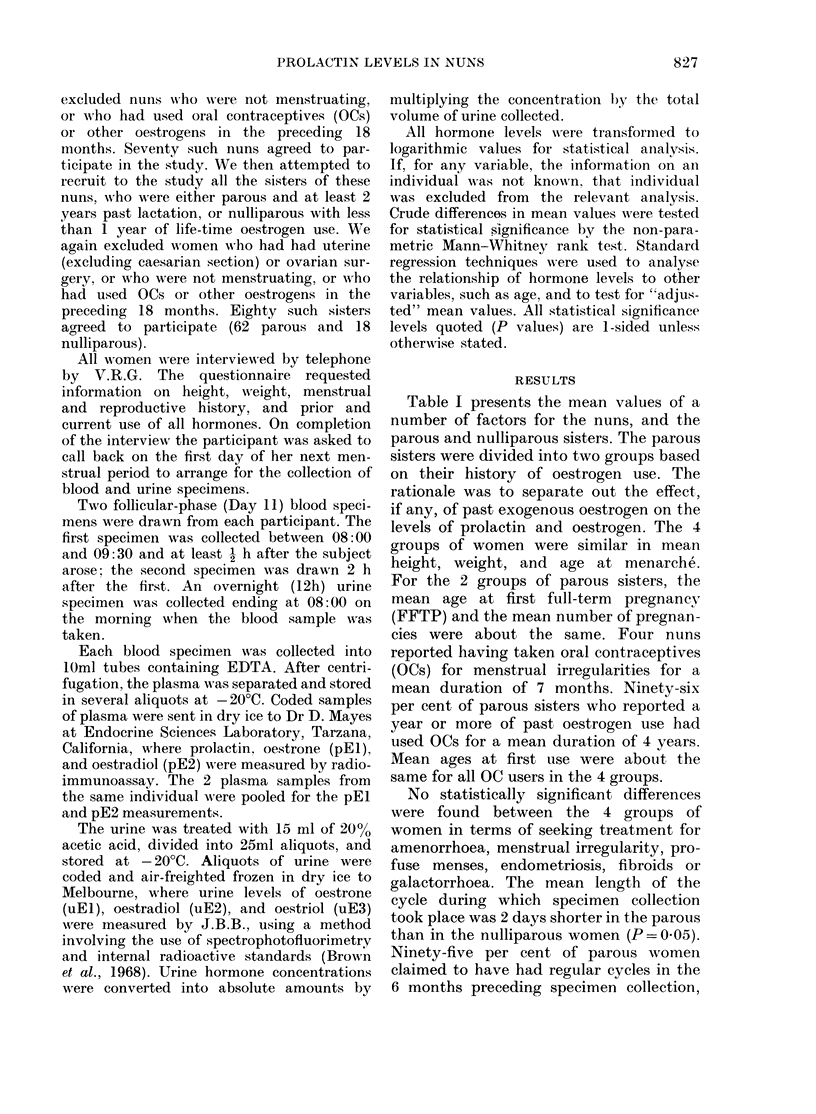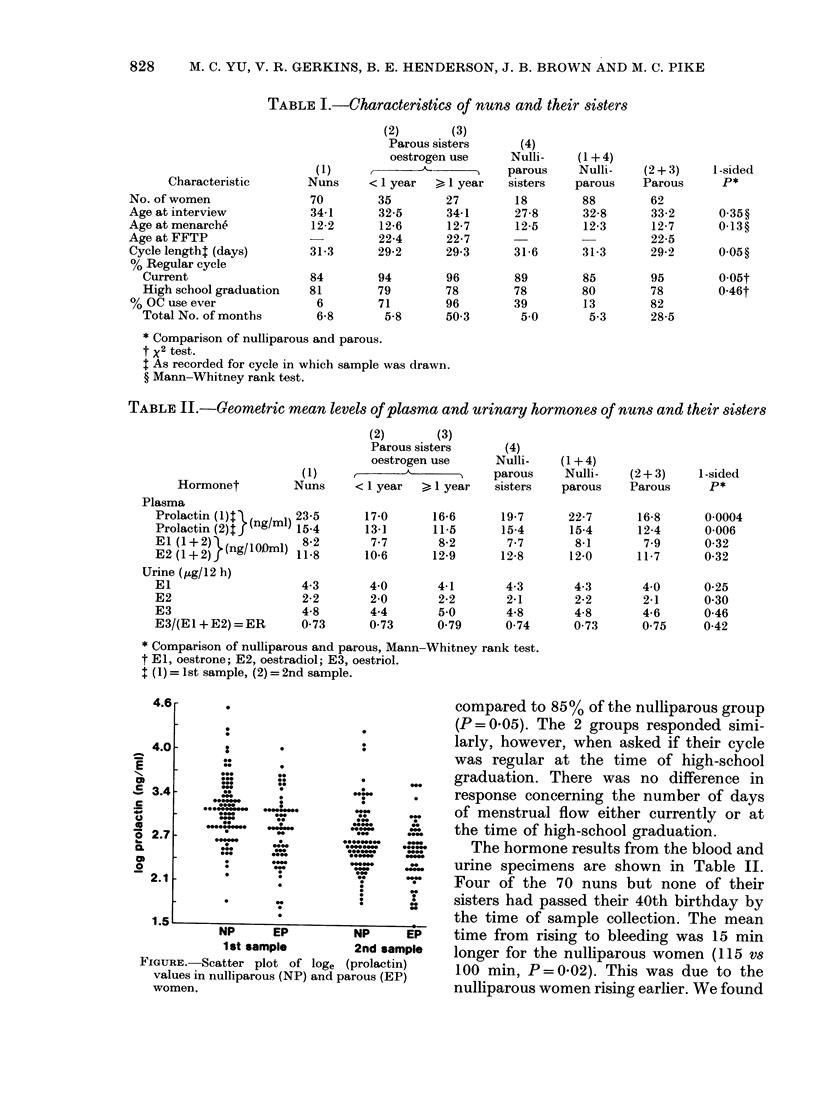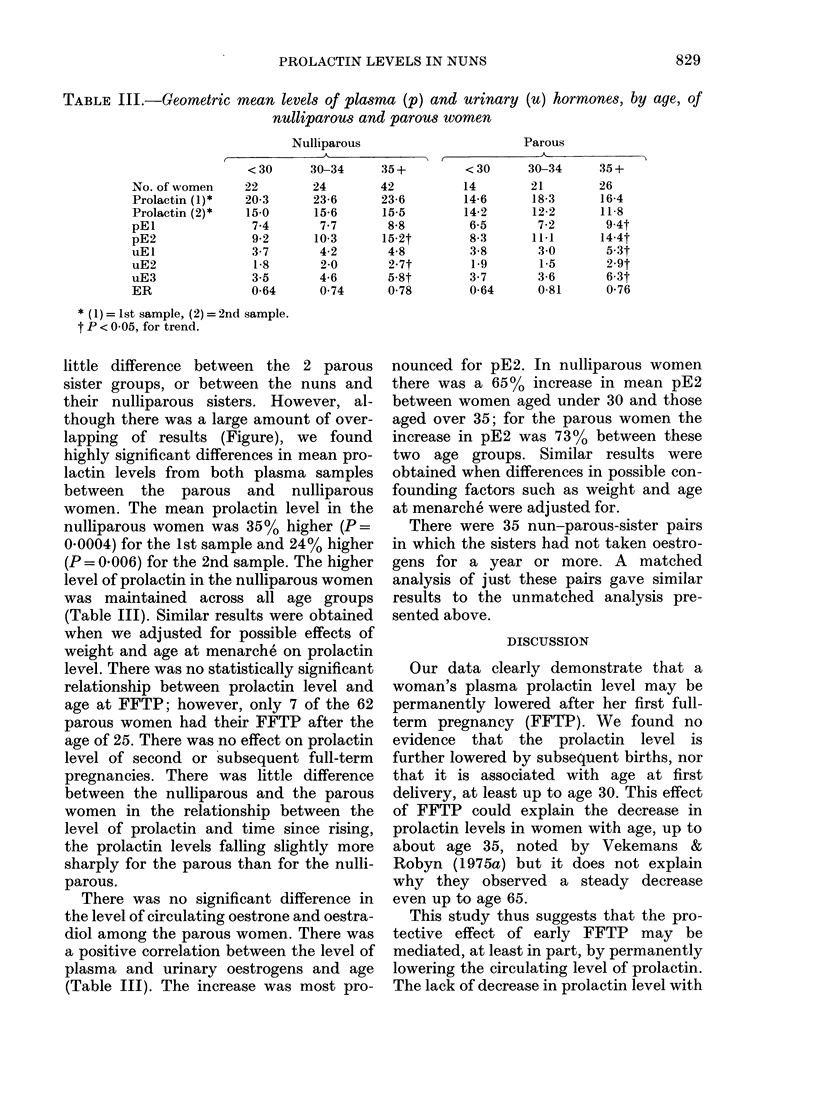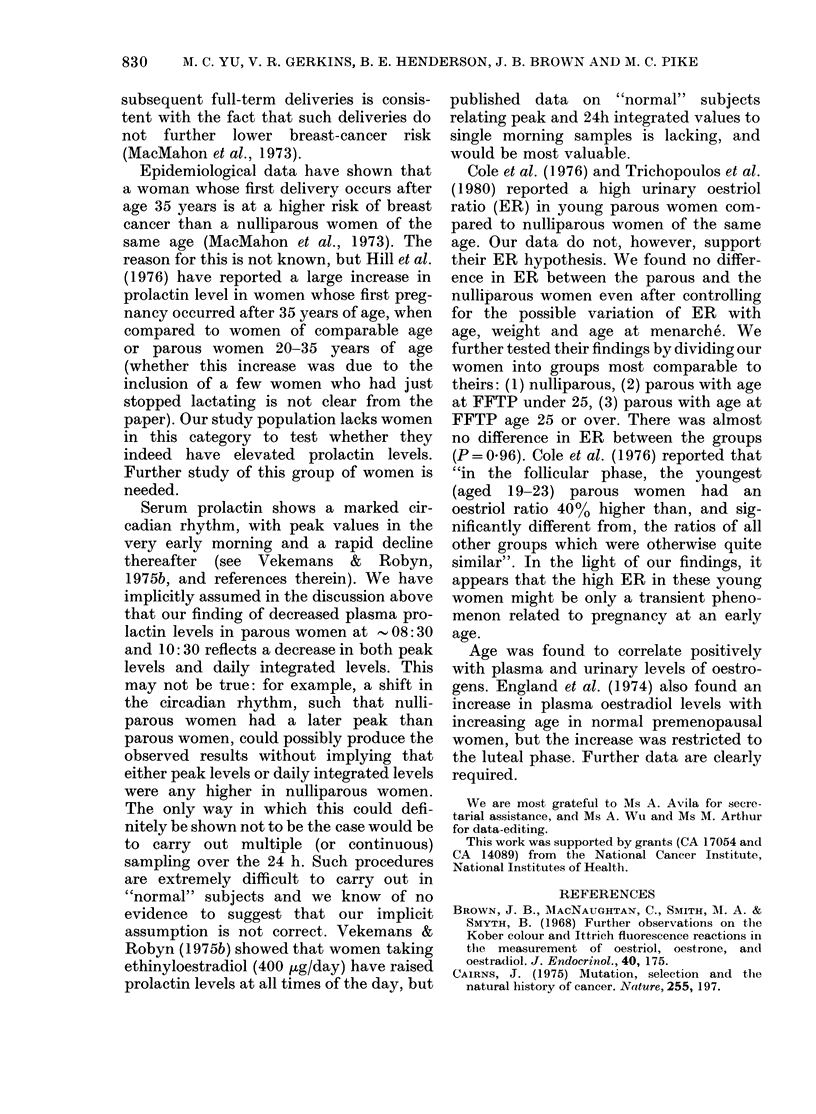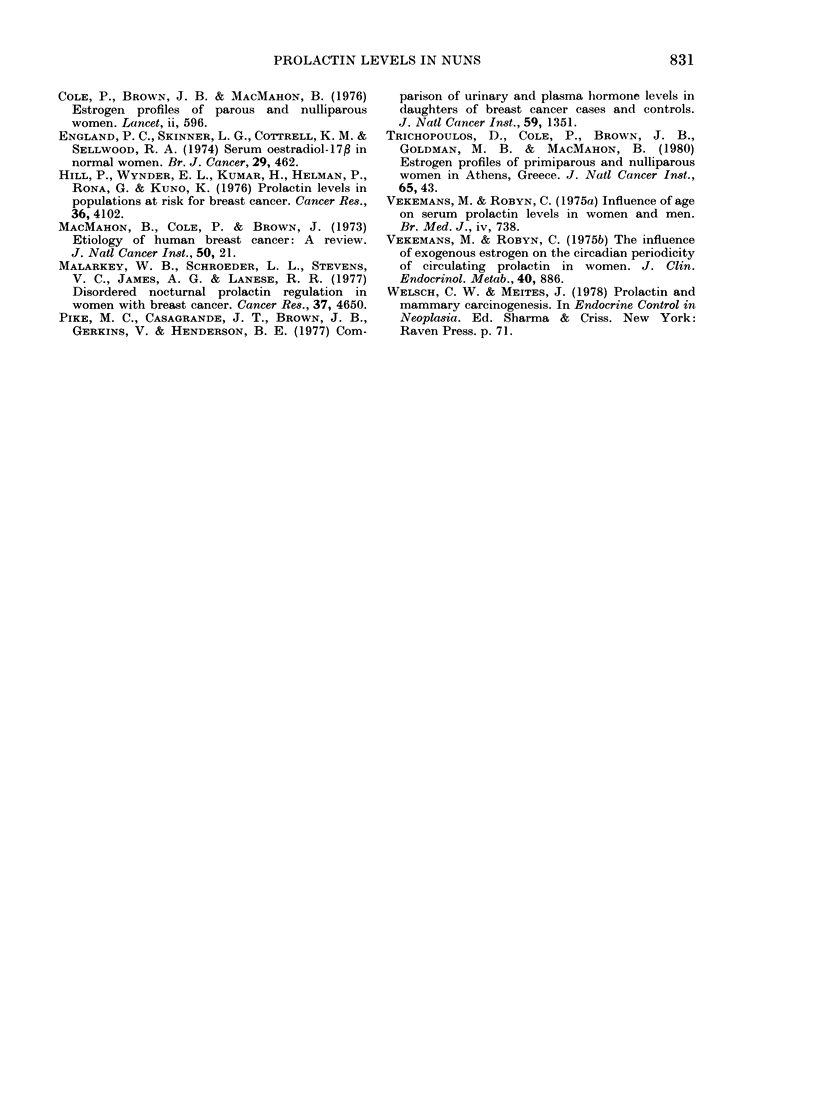# Elevated levels of prolactin in nulliparous women.

**DOI:** 10.1038/bjc.1981.121

**Published:** 1981-06

**Authors:** M. C. Yu, V. R. Gerkins, B. E. Henderson, J. B. Brown, M. C. Pike

## Abstract

Follicular-phase (Day 11) plasma prolactin, and plasma and urinary oestrogen levels of 70 nulliparous nuns were compared with those of 80 of their sisters, of whom 62 were parous. The nuns and their nulliparous sisters did not differ significantly in their prolactin and oestrogen levels. No differences in plasma oestrogens or urinary oestriol ratio were found between the parous and the nulliparous women. However, the mean prolactin level of the nuns and their nulliparous sisters was 35% higher than that of the parous women in the sample taken approximately 1 3/4 h after rising (p less than 0.0005), and 24% higher (P less then 0.01) in the 2nd sample taken 2 h later. The elevation was independent of age, weight, and age at menarché. Age at first full-term pregnancy, at least up to the age of 30, and second or subsequent full-term pregnancies had no further effect on prolactin level. This study suggests that the effect of early first full-term pregnancy in lowering breast cancer risk may be mediated, at least in part, by permanently lowering the level of circulating prolactin.


*Br. J. Cancer* (1981) **43**, 826

### ELEVATED LEVELS OF PROLACTIN IN NULLIPAROUS WOMEN

M. C. YU, V. R. GERKINS, B. E. HENDERSON, J. B. BROWN* and M. C. PIKE

*From the Department of Family and Preventive Medicine, University of Southern California School of Medicine, Los Angeles, California, U.S.A. and *Department of Obstetrics and Gynaecology, University of Melbourne, Parkville, Victoria, Australia*

Received 29 October 1980 Accepted 13 February 1981

**Summary.—Follicular-phase (Day 11) plasma prolactin, and plasma and urinary oestrogen levels of 70 nulliparous nuns were compared with those of 80 of their sisters, of whom 62 were parous. The nuns and their nulliparous sisters did not differ significantly in their prolactin and oestrogen levels. No differences in plasma oestrogens or urinary oestriol ratio were found between the parous and the nulliparous women. However, the mean prolactin level of the nuns and their nulliparous sisters was 35% higher than that of the parous women in the sample taken $\sim 1\frac{3}{4}$ h after rising ($P < 0.0005$), and 24% higher ($P < 0.01$) in the 2nd sample taken 2 h later. The elevation was independent of age, weight, and age at menarché. Age at first full-term pregnancy, at least up to the age of 30, and second or subsequent full-term pregnancies had no further effect on prolactin level. This study suggests that the effect of early first full-term pregnancy in lowering breast cancer risk may be mediated, at least in part, by permanently lowering the level of circulating prolactin.**

One of the most striking breast-cancer risk factors is age at first full-term pregnancy (FFTP): women whose FFTP is before the age of 20 have less than half the breast-cancer risk of nulliparous women (MacMahon *et al.*, 1973). Two theories have been proposed to "explain" how this protective effect of early FFTP is brought about. Cairns (1975) theorized that early FFTP would effectively reduce the number of susceptible breast "stem" cells, while Cole *et al.* (1976) proposed that the protective effect may be due to a change in the "urinary oestriol ratio". There is no evidence available to either support or refute Cairns's hypothesis, but there is some evidence that early FFTP does increase the urinary oestriol ratio (Cole *et al.*, 1976; Trichopoulos *et al.*, 1980).

Prolactin may also play a key role in determining risk of breast cancer (Hill *et al.*, 1976; Malarkey *et al.*, 1977; Pike *et al.*, 1977; Welsch & Meites, 1978) and we were struck by the finding of Vekemans & Robyn (1975*a*) in a cross-sectional study that prolactin levels decreased after age 25 in women but not in men. If this decrease with age in women is due to pregnancy, this prolactin change may be a critical part of the protective effect of early FFTP.

In this paper we describe our results in comparing the plasma prolactin, and plasma and urinary oestrogen levels of nulliparous and parous women aged 20–40 in California.

### METHODS

With the cooperation of a number of Catholic orders in California, we identified Caucasian nuns aged 20–39, who had never been pregnant and who had at least one sister aged 20–39 residing in the continental U.S. We excluded nuns who had had uterine (excluding D&C) or ovarian surgery. We also

Reprint requests and correspondence to Dr M. Yu, Department of Family and Preventive Medicine, University of Southern California School of Medicine, 2025 Zonal Avenue, Los Angeles, California 90033, U.S.A.

excluded nuns who were not menstruating, or who had used oral contraceptives (OCs) or other oestrogens in the preceding 18 months. Seventy such nuns agreed to participate in the study. We then attempted to recruit to the study all the sisters of these nuns, who were either parous and at least 2 years past lactation, or nulliparous with less than 1 year of life-time oestrogen use. We again excluded women who had had uterine (excluding caesarian section) or ovarian surgery, or who were not menstruating, or who had used OCs or other oestrogens in the preceding 18 months. Eighty such sisters agreed to participate (62 parous and 18 nulliparous).

All women were interviewed by telephone by V.R.G. The questionnaire requested information on height, weight, menstrual and reproductive history, and prior and current use of all hormones. On completion of the interview the participant was asked to call back on the first day of her next menstrual period to arrange for the collection of blood and urine specimens.

Two follicular-phase (Day 11) blood specimens were drawn from each participant. The first specimen was collected between 08:00 and 09:30 and at least $\frac{1}{2}$ h after the subject arose; the second specimen was drawn 2 h after the first. An overnight (12h) urine specimen was collected ending at 08:00 on the morning when the blood sample was taken.

Each blood specimen was collected into 10ml tubes containing EDTA. After centrifugation, the plasma was separated and stored in several aliquots at $-20$°C. Coded samples of plasma were sent in dry ice to Dr D. Mayes at Endocrine Sciences Laboratory, Tarzana, California, where prolactin, oestrone (pE1), and oestradiol (pE2) were measured by radioimmunoassay. The 2 plasma samples from the same individual were pooled for the pE1 and pE2 measurements.

The urine was treated with 15 ml of 20% acetic acid, divided into 25ml aliquots, and stored at $-20$°C. Aliquots of urine were coded and air-freighted frozen in dry ice to Melbourne, where urine levels of oestrone (uE1), oestradiol (uE2), and oestriol (uE3) were measured by J.B.B., using a method involving the use of spectrophotofluorimetry and internal radioactive standards (Brown *et al.*, 1968). Urine hormone concentrations were converted into absolute amounts by multiplying the concentration by the total volume of urine collected.

All hormone levels were transformed to logarithmic values for statistical analysis. If, for any variable, the information on an individual was not known, that individual was excluded from the relevant analysis. Crude differences in mean values were tested for statistical significance by the non-parametric Mann–Whitney rank test. Standard regression techniques were used to analyse the relationship of hormone levels to other variables, such as age, and to test for "adjusted" mean values. All statistical significance levels quoted ($P$ values) are 1-sided unless otherwise stated.

### RESULTS

Table I presents the mean values of a number of factors for the nuns, and the parous and nulliparous sisters. The parous sisters were divided into two groups based on their history of oestrogen use. The rationale was to separate out the effect, if any, of past exogenous oestrogen on the levels of prolactin and oestrogen. The 4 groups of women were similar in mean height, weight, and age at menarché. For the 2 groups of parous sisters, the mean age at first full-term pregnancy (FFTP) and the mean number of pregnancies were about the same. Four nuns reported having taken oral contraceptives (OCs) for menstrual irregularities for a mean duration of 7 months. Ninety-six per cent of parous sisters who reported a year or more of past oestrogen use had used OCs for a mean duration of 4 years. Mean ages at first use were about the same for all OC users in the 4 groups.

No statistically significant differences were found between the 4 groups of women in terms of seeking treatment for amenorrhoea, menstrual irregularity, profuse menses, endometriosis, fibroids or galactorrhoea. The mean length of the cycle during which specimen collection took place was 2 days shorter in the parous than in the nulliparous women ($P = 0{\cdot}05$). Ninety-five per cent of parous women claimed to have had regular cycles in the 6 months preceding specimen collection,

TABLE I.—*Characteristics of nuns and their sisters*

| Characteristic | (1) Nuns | (2) Parous sisters oestrogen use < 1 year | (3) Parous sisters oestrogen use ≥ 1 year | (4) Nulliparous sisters | (1+4) Nulliparous | (2+3) Parous | 1-sided $P$* |
|---|---|---|---|---|---|---|---|
| No. of women | 70 | 35 | 27 | 18 | 88 | 62 | |
| Age at interview | 34·1 | 32·5 | 34·1 | 27·8 | 32·8 | 33·2 | 0·35§ |
| Age at menarché | 12·2 | 12·6 | 12·7 | 12·5 | 12·3 | 12·7 | 0·13§ |
| Age at FFTP | — | 22·4 | 22·7 | — | — | 22·5 | |
| Cycle length‡ (days) | 31·3 | 29·2 | 29·3 | 31·6 | 31·3 | 29·2 | 0·05§ |
| % Regular cycle | | | | | | | |
| Current | 84 | 94 | 96 | 89 | 85 | 95 | 0·05† |
| High school graduation | 81 | 79 | 78 | 78 | 80 | 78 | 0·46† |
| % OC use ever | 6 | 71 | 96 | 39 | 13 | 82 | |
| Total No. of months | 6·8 | 5·8 | 50·3 | 5·0 | 5·3 | 28·5 | |

* Comparison of nulliparous and parous.
† $\chi^2$ test.
‡ As recorded for cycle in which sample was drawn.
§ Mann–Whitney rank test.

TABLE II.—*Geometric mean levels of plasma and urinary hormones of nuns and their sisters*

| Hormone† | (1) Nuns | (2) Parous sisters oestrogen use < 1 year | (3) Parous sisters oestrogen use ≥ 1 year | (4) Nulliparous sisters | (1+4) Nulliparous | (2+3) Parous | 1-sided $P$* |
|---|---|---|---|---|---|---|---|
| Plasma | | | | | | | |
| Prolactin (1)‡ (ng/ml) | 23·5 | 17·0 | 16·6 | 19·7 | 22·7 | 16·8 | 0·0004 |
| Prolactin (2)‡ (ng/ml) | 15·4 | 13·1 | 11·5 | 15·4 | 15·4 | 12·4 | 0·006 |
| E1 (1+2) (ng/100ml) | 8·2 | 7·7 | 8·2 | 7·7 | 8·1 | 7·9 | 0·32 |
| E2 (1+2) (ng/100ml) | 11·8 | 10·6 | 12·9 | 12·8 | 12·0 | 11·7 | 0·32 |
| Urine (μg/12 h) | | | | | | | |
| E1 | 4·3 | 4·0 | 4·1 | 4·3 | 4·3 | 4·0 | 0·25 |
| E2 | 2·2 | 2·0 | 2·2 | 2·1 | 2·2 | 2·1 | 0·30 |
| E3 | 4·8 | 4·4 | 5·0 | 4·8 | 4·8 | 4·6 | 0·46 |
| E3/(E1+E2) = ER | 0·73 | 0·73 | 0·79 | 0·74 | 0·73 | 0·75 | 0·42 |

* Comparison of nulliparous and parous, Mann–Whitney rank test.
† El, oestrone; E2, oestradiol; E3, oestriol.
‡ (1) = 1st sample, (2) = 2nd sample.

FIGURE.—Scatter plot of $\log_e$ (prolactin) values in nulliparous (NP) and parous (EP) women.

compared to 85% of the nulliparous group ($P = 0{\cdot}05$). The 2 groups responded similarly, however, when asked if their cycle was regular at the time of high-school graduation. There was no difference in response concerning the number of days of menstrual flow either currently or at the time of high-school graduation.

The hormone results from the blood and urine specimens are shown in Table II. Four of the 70 nuns but none of their sisters had passed their 40th birthday by the time of sample collection. The mean time from rising to bleeding was 15 min longer for the nulliparous women (115 *vs* 100 min, $P = 0{\cdot}02$). This was due to the nulliparous women rising earlier. We found

TABLE III.—*Geometric mean levels of plasma (p) and urinary (u) hormones, by age, of nulliparous and parous women*

| | Nulliparous | | | Parous | | |
|---|---|---|---|---|---|---|
| | < 30 | 30–34 | 35+ | < 30 | 30–34 | 35+ |
| No. of women | 22 | 24 | 42 | 14 | 21 | 26 |
| Prolactin (1)* | 20·3 | 23·6 | 23·6 | 14·6 | 18·3 | 16·4 |
| Prolactin (2)* | 15·0 | 15·6 | 15·5 | 14·2 | 12·2 | 11·8 |
| pE1 | 7·4 | 7·7 | 8·8 | 6·5 | 7·2 | 9·4† |
| pE2 | 9·2 | 10·3 | 15·2† | 8·3 | 11·1 | 14·4† |
| uE1 | 3·7 | 4·2 | 4·8 | 3·8 | 3·0 | 5·3† |
| uE2 | 1·8 | 2·0 | 2·7† | 1·9 | 1·5 | 2·9† |
| uE3 | 3·5 | 4·6 | 5·8† | 3·7 | 3·6 | 6·3† |
| ER | 0·64 | 0·74 | 0·78 | 0·64 | 0·81 | 0·76 |

* (1) = 1st sample, (2) = 2nd sample.

† $P < 0·05$, for trend.

little difference between the 2 parous sister groups, or between the nuns and their nulliparous sisters. However, although there was a large amount of overlapping of results (Figure), we found highly significant differences in mean prolactin levels from both plasma samples between the parous and nulliparous women. The mean prolactin level in the nulliparous women was 35% higher ($P = 0·0004$) for the 1st sample and 24% higher ($P = 0·006$) for the 2nd sample. The higher level of prolactin in the nulliparous women was maintained across all age groups (Table III). Similar results were obtained when we adjusted for possible effects of weight and age at menarché on prolactin level. There was no statistically significant relationship between prolactin level and age at FFTP; however, only 7 of the 62 parous women had their FFTP after the age of 25. There was no effect on prolactin level of second or subsequent full-term pregnancies. There was little difference between the nulliparous and the parous women in the relationship between the level of prolactin and time since rising, the prolactin levels falling slightly more sharply for the parous than for the nulliparous.

There was no significant difference in the level of circulating oestrone and oestradiol among the parous women. There was a positive correlation between the level of plasma and urinary oestrogens and age (Table III). The increase was most pronounced for pE2. In nulliparous women there was a 65% increase in mean pE2 between women aged under 30 and those aged over 35; for the parous women the increase in pE2 was 73% between these two age groups. Similar results were obtained when differences in possible confounding factors such as weight and age at menarché were adjusted for.

There were 35 nun–parous-sister pairs in which the sisters had not taken oestrogens for a year or more. A matched analysis of just these pairs gave similar results to the unmatched analysis presented above.

### DISCUSSION

Our data clearly demonstrate that a woman's plasma prolactin level may be permanently lowered after her first full-term pregnancy (FFTP). We found no evidence that the prolactin level is further lowered by subsequent births, nor that it is associated with age at first delivery, at least up to age 30. This effect of FFTP could explain the decrease in prolactin levels in women with age, up to about age 35, noted by Vekemans & Robyn (1975*a*) but it does not explain why they observed a steady decrease even up to age 65.

This study thus suggests that the protective effect of early FFTP may be mediated, at least in part, by permanently lowering the circulating level of prolactin. The lack of decrease in prolactin level with subsequent full-term deliveries is consistent with the fact that such deliveries do not further lower breast-cancer risk (MacMahon *et al.*, 1973).

Epidemiological data have shown that a woman whose first delivery occurs after age 35 years is at a higher risk of breast cancer than a nulliparous women of the same age (MacMahon *et al.*, 1973). The reason for this is not known, but Hill *et al.* (1976) have reported a large increase in prolactin level in women whose first pregnancy occurred after 35 years of age, when compared to women of comparable age or parous women 20–35 years of age (whether this increase was due to the inclusion of a few women who had just stopped lactating is not clear from the paper). Our study population lacks women in this category to test whether they indeed have elevated prolactin levels. Further study of this group of women is needed.

Serum prolactin shows a marked circadian rhythm, with peak values in the very early morning and a rapid decline thereafter (see Vekemans & Robyn, 1975*b*, and references therein). We have implicitly assumed in the discussion above that our finding of decreased plasma prolactin levels in parous women at ~08:30 and 10:30 reflects a decrease in both peak levels and daily integrated levels. This may not be true: for example, a shift in the circadian rhythm, such that nulliparous women had a later peak than parous women, could possibly produce the observed results without implying that either peak levels or daily integrated levels were any higher in nulliparous women. The only way in which this could definitely be shown not to be the case would be to carry out multiple (or continuous) sampling over the 24 h. Such procedures are extremely difficult to carry out in "normal" subjects and we know of no evidence to suggest that our implicit assumption is not correct. Vekemans & Robyn (1975*b*) showed that women taking ethinyloestradiol (400 μg/day) have raised prolactin levels at all times of the day, but published data on "normal" subjects relating peak and 24h integrated values to single morning samples is lacking, and would be most valuable.

Cole *et al.* (1976) and Trichopoulos *et al.* (1980) reported a high urinary oestriol ratio (ER) in young parous women compared to nulliparous women of the same age. Our data do not, however, support their ER hypothesis. We found no difference in ER between the parous and the nulliparous women even after controlling for the possible variation of ER with age, weight and age at menarché. We further tested their findings by dividing our women into groups most comparable to theirs: (1) nulliparous, (2) parous with age at FFTP under 25, (3) parous with age at FFTP age 25 or over. There was almost no difference in ER between the groups ($P = 0{\cdot}96$). Cole *et al.* (1976) reported that "in the follicular phase, the youngest (aged 19–23) parous women had an oestriol ratio 40% higher than, and significantly different from, the ratios of all other groups which were otherwise quite similar". In the light of our findings, it appears that the high ER in these young women might be only a transient phenomenon related to pregnancy at an early age.

Age was found to correlate positively with plasma and urinary levels of oestrogens. England *et al.* (1974) also found an increase in plasma oestradiol levels with increasing age in normal premenopausal women, but the increase was restricted to the luteal phase. Further data are clearly required.

We are most grateful to Ms A. Avila for secretarial assistance, and Ms A. Wu and Ms M. Arthur for data-editing.

This work was supported by grants (CA 17054 and CA 14089) from the National Cancer Institute, National Institutes of Health.

### REFERENCES

Brown, J. B., MacNaughtan, C., Smith, M. A. & Smyth, B. (1968) Further observations on the Kober colour and Ittrich fluorescence reactions in the measurement of oestriol, oestrone, and oestradiol. *J. Endocrinol.*, **40**, 175.

Cairns, J. (1975) Mutation, selection and the natural history of cancer. *Nature*, **255**, 197.

COLE, P., BROWN, J. B. & MACMAHON, B. (1976) Estrogen profiles of parous and nulliparous women. *Lancet*, ii, 596.

ENGLAND, P. C., SKINNER, L. G., COTTRELL, K. M. & SELLWOOD, R. A. (1974) Serum oestradiol-17$\beta$ in normal women. *Br. J. Cancer*, **29**, 462.

HILL, P., WYNDER, E. L., KUMAR, H., HELMAN, P., RONA, G. & KUNO, K. (1976) Prolactin levels in populations at risk for breast cancer. *Cancer Res.*, **36**, 4102.

MACMAHON, B., COLE, P. & BROWN, J. (1973) Etiology of human breast cancer: A review. *J. Natl Cancer Inst.*, **50**, 21.

MALARKEY, W. B., SCHROEDER, L. L., STEVENS, V. C., JAMES, A. G. & LANESE, R. R. (1977) Disordered nocturnal prolactin regulation in women with breast cancer. *Cancer Res.*, **37**, 4650.

PIKE, M. C., CASAGRANDE, J. T., BROWN, J. B., GERKINS, V. & HENDERSON, B. E. (1977) Comparison of urinary and plasma hormone levels in daughters of breast cancer cases and controls. *J. Natl Cancer Inst.*, **59**, 1351.

TRICHOPOULOS, D., COLE, P., BROWN, J. B., GOLDMAN, M. B. & MACMAHON, B. (1980) Estrogen profiles of primiparous and nulliparous women in Athens, Greece. *J. Natl Cancer Inst.*, **65**, 43.

VEKEMANS, M. & ROBYN, C. (1975*a*) Influence of age on serum prolactin levels in women and men. *Br. Med. J.*, iv, 738.

VEKEMANS, M. & ROBYN, C. (1975*b*) The influence of exogenous estrogen on the circadian periodicity of circulating prolactin in women. *J. Clin. Endocrinol. Metab.*, **40**, 886.

WELSCH, C. W. & MEITES, J. (1978) Prolactin and mammary carcinogenesis. In *Endocrine Control in Neoplasia*. Ed. Sharma & Criss. New York: Raven Press. p. 71.